# Fulminating Appendicitis

**Published:** 1901-04-06

**Authors:** 


### FULMINATING APPENDICITIS.

Dr. W. G. Richardson[1] reports three cases of acute diffuse septic peritonitis, resulting from appendicitis, in which he operated successfully. These cases were all examples of the fulminating type of appendicitis which is the most fatal of all varieties, and their successful treatment by operation is a matter on which Dr. Richardson may well be congratulated. He points out that in the initial stages they were not distinguishable from ordinary cases of appendicitis in which recovery could take place either by resolution or by limitation of the disease to the formation of an abscess, and, indeed, that the process in these fulminating cases differs from the course of events in ordinary cases, which terminate by resolution or abscess formation, only in its virulence and rapidity. In the latter cases, however, there is not the same immediate necessity for operation. It is therefore of the most urgent importance to be able to recognise these acute cases before perforation occurs, and the rule which Dr. Richardson follows is this. If the patient is seen within a few hours of the onset of the illness hot fomentations are applied to the abdomen. Dr. Richardson then waits and sees what progress is made, and as long as steady improvement continues waiting is persevered in. But by improvement he means improvement in *all* the symptoms. All the symptoms should subside together; the temperature should fall, the pulse should become less frequent, local pain and tenderness should grow less, and the patient should look better. But if in a very acute case *all* the symptoms have not improved at the end of 24 hours, then one should operate. Doubtless if such a rule be followed a number of cases will be operated on in which, although the appendix is on the point of giving way, it is yet guarded by adhesions. But against this must be placed the risk of waiting, and the possibility of abscess formation, and, above all, the simplicity and safety of operating at this stage must be taken into account.

[1] Lancet, Mar. 23.

